# Dietary Patterns and The Outcomes of Assisted Reproductive Techniques in Women with Primary Infertility: A Prospective Cohort Study 

**DOI:** 10.22074/ijfs.2019.5373

**Published:** 2018-10-02

**Authors:** Maryam Jahangirifar, Mahboubeh Taebi, Mohammad Hossein Nasr-Esfahani, Gholamreza Askari.

**Affiliations:** 1 Nursing and Midwifery Care Research Center, Isfahan University of Medical Sciences, Isfahan, Iran; 2 Department of Midwifery and Reproductive Health, Woman's Health Research Center, Isfahan University of Medical Sciences, Isfahan, Iran; 3 Department of Cellular Biotechnology, Cell Science Research Center, Royan Institute for Biotechnology, ACECR, Isfahan, Iran; 4 Department of Community Nutrition, School of Nutrition and Food Science, Isfahan University of Medical Sciences, Isfahan, Iran

**Keywords:** Assisted Reproductive Technique, *In vitro* Fertilization, Infertility, Nutrition

## Abstract

**Background:**

Infertility is one of the most common challenges that women in reproductive age would encounter today. The maternal nutritional status could be a determinant of oocyte quality and embryonic growth. This study was conducted to assess the relationship between dietary patterns and reproductive outcomes in infertile women.

**Materials and Methods:**

This prospective cohort study was conducted on 140 women with primary infertility who had referred to Isfahan Fertility and Infertility center, Isfahan, Iran. The average number of total oocytes and metaphase II oocytes, the fertilization rate, the ratio of good and bad quality embryo and biochemical and clinical pregnancy were considered as the outcomes of assisted reproductive techniques (ART). A 168-item food frequency questionnaire was used for estimating the dietary intakes during the last year. Factor analysis was used for identifying the dietary patterns and analysis of variance (ANOVA), analysis of covariance (ANCOVA), chi-square, and logistic regression analysis were used for assessing the relation between dietary patterns and ART’s outcomes.

**Results:**

Three major dietary patterns (the healthy, western and unhealthy diet) were identified. Women with high adherence to the “healthy diet” had a higher educational level and were employed. There was a significant increase in the average number of total oocytes (P-trend=0.009) and metaphase II oocytes (P-trend=0.006) in the third tertile of “healthy diet” compared to the first tertile. Also, women with high adherence to the second tertile of “unhealthy” diet compared to the first tertile, had a significantly lower chance of getting pregnant [odds ratio (OR): 0.14, 95% confidence interval (CI): 0.3-0.7].

**Conclusion:**

Nutrition status could affect infertility treatment outcomes. Greater adherence to the healthy diet may enhance oocyte quality and quantity. Unhealthy diet could adversely affect the chance of getting pregnant.

## Introduction

Infertility is defined as the inability to conceive a clinical pregnancy after 12 months of unprotected intercourse (1). It is one of the most common challenges faced by women of reproductive age today (2). Infertility is estimated to affect between 8 to 12% of reproductive-aged couples worldwide (3) and the overall mean of infertility in Iran is 13.2% (4).

According to the Society for Assisted reproductive technology (ART), of 39,573 assisted reproductive cycles performed in the United States clinics among women younger than the age of 35 in 2014, only 37.1% of fresh nondonor ART cycles have resulted in live births (5). So it can be concluded that there is more room for research for improving ART outcomes. One focus area for researchers could be the relation between intra-follicular environment and oocyte quality. Indeed, a viable pregnancy is highly related to the oocyte quality which is related to the follicular environment (6). In this regard, it has been shown that oocyte growth is sensitive to changes in the follicular environment especially nutrient changes. Variance in maternal nutrition can have a significant effect on the metabolic activity of oocytes, oocyte quality and the resultant embryo and its development (7). Therefore, preconception maternal nutritional status is shown to be an important determinant of embryonic and fetal growth (8). Despite these conclusions, the effect of preconception interventions such as nutritional diet on fertility and pregnancy outcomes is unclear (9) and human studies in this field are few.

However, animal studies have shown that deficiencies or excesses in a range of macro- and micronutrients during pre-conception period can lead to impairments in fertility and fetal development and affect the long-term offspring health (10). Improper nutrition is a growing problem worldwide and it is estimated that up to 57.8% of the world’s adult population (3.3 billion people) could be either overweight or obese by 2030 (11).

There are many studies that examine the impact of micro- and macronutrients on reproductive health and pregnancy outcomes (7). But recently, there is a new interest in how the overall diet can affect reproductive outcomes especially in infertile women. This approach is more realistic because it reflects the way in which food is consumed and takes into account complicated interactions between nutrients in the diet (12). Because of the association between nutrient intakes and dietary patterns, the effect of a single nutrient may be confounded by the effect of dietary patterns. Also the effect of multiple nutrients rather than a single nutrient could be big enough to detect (13). Human studies on whether or not dietary patterns can affect *in vitro* fertilization (IVF) and intracytoplasmic sperm injection (ICSI) outcomes are few and the relation between these two parameters is not clear. So there is a potential need for more research on the relation between dietary patterns and infertility treatment outcomes.

The aim of the present study was to assess the relation between dietary patterns and reproductive outcomes in women with primary infertility seeking ART.

## Materials and Methods

The present study was a prospective cohort study that was performed at Isfahan Fertility and Infertility center, Isfahan, Iran. A simple sampling design was used. The following formula was used for calculating the sample size:

$$n=\frac{{\left(Z_{1}+Z_{2}\right)}^{2}.2P(1-P)}{d^{2}}$$

Z_1_=1.6

Z_2_=0.84

P=40% (an estimate of pregnancy rate in fresh nondonor

IVF cycles in each tertile (14)

d=0.6P

The sample size for each tertile was estimated to be 65 women plus a 10% sample dropping. So 217 participants were included in the study.

Between August 2015 and January 2016, 217 women with primary infertility aged between 20-45 years who were undergoing suppression protocol for IVF/ICSI, were invited to participate in this study. The inclusion criteria were having female infertility (idiopathic or ovarian infertility), not having significant changes in diet during the last 3 months or having a specific diet, not consuming alcohol and not smoking, not having a history of diseases affecting metabolism including diabetes, galactosemia, maple syrup urine disease (MSUD), phenylketonuria (PKU), inflammatory bowel disease, celiac disease, chronic pancreatitis, nephrotic syndrome, acute and chronic kidney failure, dialysis, hypothyroidism and hyperthyroidism (15, 16) and not using drugs that affect the metabolism of macronutrients including antihyperglycemic and lipid-altering agents (metformin hydrochloride consumption considered as confounding variable). Exclusion criteria were deciding to hire a surrogate mother, having male factor infertility, and discontinuing cooperation in the study or ART process.

All participants filled out the 168-item food frequency questionnaire (FFQ) and the short form of international physical activity questionnaire (IPAQ). Also their demographic characteristics were recorded. Body mass index (BMI) was calculated as weight in kg divided by the square of height in meters (kg/m^2^). The waist circumference was measured with a non-stretch tape to the nearest 0.1 cm between the lowest rib margin and the iliac crest with minimal clothing (17).

In the laboratory, the researcher recorded the total number of retrieved oocytes and the number of Metaphase II oocytes. Then IVF/ICSI procedure was conducted based on the standard protocols using G-V series media form VitroLife (18). Fertilization rate was defined as the ratio of zygotes with two pronuclei observed 16-18 hours after insemination divided by the number of inseminated oocytes (19). Embryos were scored using a four-point scale on day 3. One point was assigned to all cleaved embryos, and an additional point was added for each of the following features: the absence of fragmentation (or fragmentation involving 25% of embryonic surface), the absence of irregularities in blastomere size or shape, and 8-cell stage on day 3 (20). Therefore, embryo score ranged from 2 to 4. Embryos with a score of 2 were considered as a bad quality embryo, and a score of 4 indicated good quality embryo. Their rates were calculated by dividing the number of the bad or good embryos by the total number of embryos. Biochemical pregnancy was confirmed by βHCG test, 12 days after embryo transfer (21). Clinical pregnancy was defined as the presence of one or more gestational sacs during transvaginal scan 3 weeks after embryo transfer (22).

### Questionnaires

The 168-item FFQ was validated in a previous study (23) for estimating dietary intakes and their frequency of consumption for each food during the last year. The amount of each food item was converted into gram using household scale guide and calculated for one day. Food items were categorized into food groups according to the similarity of their nutrient composition and logged into SPSS software. IPAQ was used for assessing daily physical activity as a confounder. The physical activity was computed by weighting each type of activity by its energy requirements defined in MET-Min/Day. Then it was converted to MET-h/week and reported. This questionnaire has been validated in previous studies (24).

### Ovulation induction

In this study, suppression protocol was used. On the second day of the last menstrual period, when no ovarian cyst was observed in a transvaginal ultrasound scan, ovarian stimulation was commenced with Gonal-F (Serono, Switzerland) in combination with Menogon (Ferring, Germany). Serial ultrasound scans were carried out and when the dominant follicle reached the size of 13-14 mm, gonadotropin-releasing hormone (GnRH) antagonist was administered daily. Ovulation was triggered with 10,000 IU hCG, when the size of dominant follicles reached 17-18 mm. After 36 hours, transvaginal oocyte retrieval was carried out.

### Ethical considerations

All participants were informed of the details of the study and were allowed to leave the study at any time. Informed consent was obtained from all the women who agreed to participate in the study. The study was approved by the Ethics Committee of the Isfahan University of Medical Sciences.

### Statistical analysis

To identify major dietary patterns, factor analysis was used with oblique transformation. Factors were retained using the Scree test, if their eigenvalues were >1.5. Three factors were selected as major dietary patterns based on the Scree test and eigenvalues. Dietary patterns were labeled based on the previous knowledge about nutrition and according to the food groups with highest factor loading (25). All the participants were in all the dietary patterns and, based on their personal score, were divided into tertiles; meaning that, those who had the lowest and highest intake of foods in each dietary pattern were placed respectively in the first and third tertiles. Significant differences in general characteristics across tertiles were assessed by ANOVA for continuous variables. Chi-square test was used for assessing the distribution of categorical variables across tertiles. ANOVA and ANCOVA analysis were used to determine the association of dietary patterns with fertility markers including the average number of total oocytes, the average number of metaphase II oocyte and the ratio of good and bad quality embryo. All models were adjusted for age, marriage age, BMI, waist circumference, supplement consumption, metformin consumption and physical activity. Logistic regression analysis was used (with covariates as above) to calculate adjusted odds ratios (OR) and 95% confidence interval (CI) for assessing the association of dietary patterns with biochemical and clinical pregnancy.

To perform all the statistical analyses, SPSS software version 20.0 (Armonk, NY: IBM Corp) was used. P<0.05 was considered significant.

## Results

A total of 217 infertile women participated in this study. Because of discontinuing the treatment due to ovarian hyperstimulation syndrome, unwillingness to continue the study, and diagnosing male factor infertility in the next steps of research, 77 cases were excluded from the study.

Three major dietary patterns were identified among 140 women. The first dietary pattern was named the “healthy diet” and included high consumption of fruits, nuts, vegetables, red and white meat, dairy, green olive, cream, and legume. The second dietary pattern was labeled as the “western diet” and was comprised of high consumption of sweet drinks, sweets, caffeinated drinks, potatoes, fast foods, whole grains, refined grains, liquid oils, and salt. The third dietary pattern was called the “unhealthy diet” and contained high consumption of mayonnaise, butter, egg, junk foods and solid oils (Table 1). Demographic characteristics of the participants according to the tertiles of dietary patterns are presented in Table 2. The results showed that women with high adherence to the “healthy diet” had a higher educational level and were employed.

**Table 1:** Factor loading for food groups of the three dietary patterns identified from food frequency questionnaire (FFQ) in 140 infertile women


Food groups	Healthy	Western	Unhealthy
	
	dietary pattern		

Fruits	0.750	-	-
Nuts	0.672	-	-
Vegetables	0.597	-	-
Meat	0.535	-	-
Dairy	0.418	-	-
Green olive	0.443	-	-
Cream	0.272	-	-
Legume	0.142	-	-
Sweet drinks	-	0.782	-
Sweets	-	0.519	-
Caffeinated drinks	-	0.480	-
Potato	-	0.416	-
Fast foods	-	0.344	-
Whole grain	-	-0.334	-
Refined grain	-	0.303	-
Liquid oil	-	0.298	-
Salt	-	0.237	-
Mayonnaise sauce	-	-	0.777
Butter	-	-	0.738
Egg	-	-	0.509
Junk foods	-	-	0.320
Solid oil	-	-	0.279
Variance explained (%)	12.679	8.892	7.133


**Table 2:** Demographic characteristics by tertiles of the dietary patterns


Characteristic	n=140	Tertile of healthy pattern				Tertile of western pattern				Tertile of unhealthy pattern			
		T_1_	T_2_	T_3_	P value^a^	T_1_	T_2_	T_3_	P value	T_1_	T_2_	T_3_	P value
		n=46	n=47	n=47		n=46	n=47	n=47		n=46	n=47	n=47	

Age (Y), mean ± SD	32.4 ± 5.2	32.4 ± 5.6	33.2 ± 5.2	31.6 ± 4.7	0.294	31.3 ± 5	33.6 ± 5	32.3 ± 5.4	0.106	32.7 ± 4.7	33 ± 5.3	31.5 ± 5.6	0.324
BMI (kg/m^2^), mean ± SD	28.1 ± 4.9	28.6 ± 5.5	28.3 ± 4.4	27.2 ± 4.6	0.338	28.2 ± 5.2	28.1 ± 4.5	27.9 ± 4.9	0.969	28.2 ± 5	28.2 ± 4	27.7 ± 5.6	0.875
Waist circumference (cm), mean ± SD	83.4 ± 10.4	84.3 ± 11.7	83.6 ± 9.2	82.3 ± 10.1	0.642	84 ± 10	83.5 ± 10	82.7 ± 11.3	0.842	84.1 ± 10.1	82.5 ± 7.9	83.6 ± 12.8	0.747
Education, count (%)					0.003				0.357				0.864
Under diploma	37 (26.4)	21 (45.7)	11 (23.4)	5 (10.6)	-	14 (30.4)	9 (19.1)	14 (29.8)	-	10 (21.7)	15 (31.9)	12 (25.5)	-
Diploma	45 (32.1)	13 (28.3)	16 (34)	16 (34)	-	11 (23.9)	20 (42.6)	14 (29.8)	-	16 (34.8)	14 (29.8)	15 (31.9)	-
Academic	58 (41.4)	12 (26.1)	20 (42.6)	26 (55.3)	-	21 (45.7)	18 (38.3)	19 (40.4)	-	20 (43.5)	18 (38.3)	20 (42.6)	-
Employment status, count (%)					0.033				0.207				0.423
Housewife	110 (78.6)	42 (91.3)	35 (74.5)	33 (70.2)	-	39 (84.8)	38 (80.9)	33 (70.2)	-	37 (80.4)	34 (72.3)	39 (83)	-
Employed	30 (21.4)	4 (8.7)	12 (25.5)	14 (29.8)	-	7 (15.2)	9 (19.1)	14 (29.8)	-	9 (19.6)	13 (27.7)	8 (17)	-
The cause of infertility, count (%)					0.523				0.111				0.482
Ovarian	105 (75)	33 (71.7)	38 (80.9)	34 (72.3)	-	39 (84.8)	35 (74.5)	31 (66)	-	34 (73.9)	33 (70.2)	38 (80.9)	-
Idiopathic	35 (25)	13 (28.3)	9 (19.1)	13 (27.7)	-	7 (15.2)	12 (25.5)	16 (34)	-	12 (26.1)	14 (29.8)	9 (19.1)	-


^a^; P value from One-way analysis of variance for continuous quantitative variables and from Chi-square test for categorical variables.

**Table 3:** Comparison of the assisted reproductive technology outcomes by tertiles of the dietary patterns in infertile women


Variables	Helthy pattern				Western pattern				Unhelthy pattern			
	T_1_	T_2_	T_3_	P value^c^	T_1_	T_2_	T_3_	P value	T_1_	T_2_	T_3_	P value

The average number of total oocyte												
Model_1_^a^ (mean ± SD)	7.1 ± 6.1	10.6 ± 8.6	12.2 ± 9	0.009	12 ± 9.3	8.7 ± 6.7	9.2 ± 8.3	0.119	9.8 ± 7.9	9.8 ± 8.8	10.4 ± 8.1	0.929
Model_2_^b^ (mean ± SE)	7.4 ± 1.2	11.1 ± 1.2	11.4 ± 1.3	0.053	12.1 ± 1.2	9.5 ± 1.1	8.4 ± 1.2	0.088	9.8 ± 1.2	10.5 ± 1.2	9.6 ± 1.2	0.837
The average number of metaphase II oocyte												
Model_1_ (mean ± SD)	6.1 ± 5.3	8.6 ± 6.8	10.6 ± 7.9	0.006	10 ± 7.4	7.5 ± 6.3	7.9 ± 7.1	0.184	8.6 ± 7	8.2 ± 7.6	8.6 ± 6.4	0.956
Model_2_ (mean ± SE)	6.2 ± 1	8.9 ± 1	10.2 ± 1.1	0.034	9.9 ± 1	8.1 ± 1	7.4 ± 1	0.225	8.6 ± 1	8.8 ± 1	7.9 ± 1	0.824
The fertilization rate												
Model_1_ (mean ± SD)	0.61 ± 0.4	0.7 ± 0.3	0.7 ± 0.2	0.168	0.7 ± 0.3	0.7 ± 0.3	0.7 ± 0.3	0.776	0.7 ± 0.3	0.7 ± 0.3	0.6 ± 0.3	0.649
Model_2_ (mean ± SE)	0.6 ± 0.05	0.7 ± 0.05	0.7 ± 0.05	0.310	0.7 ± 0.05	0.7 ± 0.05	0.7 ± 0.05	0.545	0.7 ± 0.05	0.7 ± 0.05	0.6 ± 0.05	0.536
The ratio of good quality embryo												
Model_1_ (mean ± SD)	0.2 ± 0.3	0.2 ± 0.2	0.2 ± 0.3	0.705	0.2 ± 0.3	0.2 ± 0.3	0.2 ± 0.3	0.870	0.2 ± 0.3	0.2 ± 0.3	0.2 ± 0.3	0.433
Model_2_ (mean ± SE)	0.2 ± 0.05	0.2 ± 0.04	0. 2 ± 0.05	0.874	0.2 ± 0.04	0.2 ± 0.04	0.2 ± 0.04	0.656	0.2 ± 0.04	0.2 ± 0.04	0.2 ± 0.04	0.352
The ratio of bad quality embryo												
Model_1_ (mean ± SD)	0.2 ± 0.3	0.4 ± 0.3	0.3 ± 0.3	0.159	0.3 ± 0.3	0.3 ± 0.3	0.3 ± 0.3	0.559	0.3 ± 0.3	0.3 ± 0.3	0.3 ± 0.3	0.733
Model_2_ (mean ± SE)	0.3 ± 0.05	0.4 ± 0.05	0.3 ± 0.05	0.425	0.3 ± 0.05	0.3 ± 0.05	0.4 ± 0.05	0.653	0.3 ± 0.05	0.3 ± 0.05	0.3 ± 0.05	0.923
The biochemical pregnancy [OR (IC)]^d^												
Model_1_ (mean ± SD)	1	1.5 (0.4-5.3)	1.1 (0.3-4.2)	0.816	1	1.4 (0.3-5.5)	1.4 (0.4-5.1)	0.858	1	0.14 (0.3-0.7)	0.72 (0.2-2.6)	0.036
Model_2_ (mean ± SE)	1	1.3 (0.3-5.8)	1.3 (0.3-6.7)	0.914	1	1.8 (0.3-9.4)	1.1 (0.2-5.9)	0.749	1	0.09 (0.01-0.6)	0.9 (0.1-5.9)	0.022
The clinical pregnancy[OR (IC)]												
Model_1_ (mean ± SD)	1	1.5(0.4-5.3)	1.1(0.3-4.2)	0.816	1	1.4(0.3-5.5)	1.4(0.4-5.1)	0.858	1	0.14 (0.3-0.7)	0.72(0.2-2.6)	0.036
Model_2_ (mean ± SE)	1	1.3(0.3-5.8)	1.3(0.3-6.7)	0.914	1	1.8(0.3-9.4)	1.1(0.2-5.9)	0.749	1	0.09 (0.01-0.6)	0.9(0.1-5.9)	0.022


Model_1_^a^; Crude, Model_2_^b^; Adjusted for age, marriage age, BMI, waist circumference, physical activity, total energy intake, supplement consumption, duration of metformin consumption, ^c^; P trends from ANOVA analysis for model_1_ and from ANCOVA analysis for model_2_ in quantitative variables and p trends from logistic regression analysis for qualitative variables, and ^d^; OR (CI): Odds ratio and 95% interval confidence calculated by logistic regression analysis.

There was a significant increase in the average number of total oocytes (P-trend=0.009) and metaphase II oocytes (P-trend=0.006) in the third tertile of “healthy diet” compared to the first tertile. After adjusting for confounding variables, these relations remained significant for the average number of total oocytes (P-trend=0.053) and metaphase II oocytes (P-trend=0.034). But the “western diet” and the “unhealthy diet” were not associated with the average number of total and metaphase II oocytes. Women with high adherence to the second tertile of “unhealthy diet” compared to the first tertile, had a significantly lower chance of getting pregnant (chemical and clinical pregnancy) (model1: OR: 0.14, 95% CI: 0.3-0.7, model2: OR: 0.09, 95% CI: 0.01-0.6). None of these dietary patterns were associated with the fertilization rate and the ratio of good and bad quality embryo (Table 3).

## Discussion

The present study assessed the relation between dietary patterns and outcome of ART in infertile women undergoing IVF/ICSI procedure. The first dietary pattern included high consumption of fruits, nuts, vegetables, red and white meat, dairy, green olive, cream, and legume. Intake of fruits, nuts, and vegetables was outstanding. So this dietary pattern was called as the “healthy diet” (26). The second dietary pattern comprised of high consumption of sweet drinks, sweets, caffeinated drinks, potatoes, oil, fast food, refined grains, whole grains and salt. The highest factor loadings belonged to sweet drinks, sweets, potato, refined grains, and salt, so it was labeled as the “western diet” (27). The third dietary pattern was rich in unhealthy food groups such as mayonnaise sauce, butter, junk foods and solid oils and was named the “unhealthy diet”.

Based on the results, women with the highest “healthy diet” score had a higher educational level and were employed. So higher education and being employed were predictors of a healthy diet. This relation has been seen in previous studies too (28).

Present findings showed that dietary patterns were related to some ART outcomes. The average number of total and metaphase II oocytes was increased significantly in women with high adherence to the “healthy diet”. These relations were significant even after taking confounding variables into account.

A randomized clinical trial showed that having a healthy diet rich in fruits and vegetable, like dietary approach for stopping hypertension (DASH), can have a positive effect on total antioxidant capacity (TAC). In this study, 60 women with polycystic ovary syndrome (PCOS) were equally randomized into DASH diet (being rich in fruits, vegetables, whole grains and low-fat dairy products, as well as low in saturated fats, cholesterol, refined grains and sweets) and control groups. After a 12-week follow-up, the DASH diet group had an increase in TAC (29). In another study it was shown that the DASH diet is associated with an increase in plasma TAC in obese patients with PCOS (30).

In this study, the “healthy diet” was richer in vegetable, fruits, and nuts. These food groups contained a high level of antioxidants (31). Antioxidants in fruits and vegetables may significantly contribute to an antioxidant capacity increase in plasma (32). The TAC levels may have a positive effect on the percentage of metaphase ΙΙ stage oocyte and oocyte quality (33).

No relation was observed between fertilization rate and all three dietary patterns. Despite the fact that male factor infertility had been excluded in this study, environmental factors such as nutrition and job might affect the sperm quality and fertilization.

In the present study, the chance of getting pregnant was decreased significantly in women who had more adherence to the “unhealthy diet”. This diet contained foods high in saturated fatty acids like mayonnaise sauce, butter, and solid oils. A previous study reported that dietary fatty acid content reflects in the fatty acid profile of follicular fluid (34). Beneficial effects have been considered for unsaturated fatty acids and deleterious effects for saturated fatty acids in elevated concentrations (35). Haggarty et al. (36) showed that human embryos which were developed beyond the four-cell stage had a higher ratio of unsaturated compared to saturated fatty acids. Also Luzzo et al. (37) showed that in mice with a high-fat diet the percentage of embryos developing to a blastocyst stage was significantly lower than the control group (~50% vs. 65%). So there was a lower chance to go on to implant and establish pregnancy similar to the control groups.

No relation was found between the healthy and western diets and the chance for getting pregnant.

A prospective cohort study in the Netherlands on 161 subfertile couples undergoing IVF/ICSI treatment showed that the Mediterranean diet, as a healthy diet, has a 40% increased probability of achieving pregnancy after IVF/ICSI treatments (38). Also findings of a case-control study conducted on 485 women with difficulty getting pregnant and 1,669 age-matched controls who had at least one child showed that there was a lower risk of difficulty getting pregnant in the highest quartile of adherence to the Mediterranean-type pattern compared to the lowest quartile. But having western dietary pattern did not have a statistically significant relation with consulting a physician because of difficulty in getting pregnant (39).

Some limitations in the present study might cause these null findings. Although male factor infertility was excluded, environmental factors like nutrition were uncontrollable and could affect fertilization and establishment of pregnancy. Also we could not distinguish chromosomally abnormal embryos from normal. These abnormal embryos might fail to implant and be less affected by the diet (40). So chromosomal abnormalities in blastocysts could nullify the results. Finally, despite controlling various confounders in the present analysis, residual confounders, which were unknown, could not be excluded.

One strength of the present study was its prospective cohort design so multiple assisted reproductive outcomes could have been evaluated. Also, the current study is the first follow-up study that was performed in countries undergoing nutrition transition. For assessing dietary intakes, a validated FFQ which reflects Iranian food consumption was used and filled out by an experienced interviewer. Also, many confounder variables such as age, marriage age, BMI, waist circumference, physical activity, total energy intake, supplement consumption and duration of metformin consumption were adjusted.

Since the number of questions was too much, it is probable that some of the participants had not answered the food frequency questionnaire accurately and this was one of the limitations of the present study. Although a validated FFQ was used and filled out by an experienced interviewer, measurement bias was unavoidable and might underestimate or overestimate dietary intakes.

Results of the present study suggested that the average number of total and metaphase II oocytes were significantly higher in women with high adherence to “healthy diet” than the others, with low adherence. Also having “unhealthy diet” decreased the chance of getting pregnant after IVF or ICSI procedure.

## Conclusion

The present study showed that having a “healthy diet” had a positive effect and could cause an increase in the average number of total and metaphase II oocytes and an “unhealthy diet” could decrease the chance of getting pregnant. The fertilization rate and the ratio of good and bad quality embryo were not affected by any of the dietary patterns. The results showed that nutrition status could affect infertility treatment outcomes. So nutritional interventions before attempting the infertility treatments could improve outcomes, reduce costs, and increase the mental and fertility health in couples. Considering the small number of conducted studies, it is suggested to perform more investigations on this issue to elucidate this relation and evaluate nutritional effects on reproductive health especially in infertile women.
